# From Environmental Exposure to Fixed Somatic Belief: Delusional Parasitosis at the Boundary of Trauma and Obsession

**DOI:** 10.1192/bjo.2026.11875

**Published:** 2026-06-30

**Authors:** Emeka Onochie, Saminathan Anand

**Affiliations:** Lincolnshire Partnership NHS Foundation Trust, Lincoln, United Kingdom

## Abstract

**Aims:**

Delusional parasitosis is defined by a persistent belief of infestation despite absence of objective evidence and is classified as a somatic delusional disorder in ICD-11 and DSM-5-TR. It often arises where plausible environmental exposure interacts with affective vulnerability and pre-existing anxiety traits, blurring the line between obsessional preoccupation and psychotic conviction. Such cases provide insight into how real-world threats can crystallise into enduring delusional beliefs, exposing the limitations of rigid diagnostic categories.

**Methods:**

Mrs X, a 65-year-old woman with a history of obsessive–compulsive disorder, agoraphobia, and depression, was referred for psychiatric assessment because of persistent concerns that insects inhabited her skin, clothing, and living environment. Her symptoms began following a suspected flea exposure while living in Cornwall, which she experienced as highly distressing. Despite relocation, repeated cleaning and environmental interventions, and multiple medical reviews showing no evidence of infestation, her conviction persisted.

She described ongoing sensations of particles entering her skin, ears, and face, with burning and itching, worsened at night. These experiences became central to daily life. She discarded most personal belongings, slept on inflatable mattresses, engaged in repetitive washing and decontamination rituals, and spent hours documenting “evidence” through photographs, videos, and microscopic examination. Progressive social withdrawal, disrupted sleep, reduced appetite, and secondary low mood developed as her preoccupation intensified.

Examination revealed only healing excoriations and minor skin dryness without objective signs of infestation or systemic illness. Mental state assessment showed anxiety, labile affect, circumstantial speech, and fixed somatic beliefs with limited insight. There were no hallucinations or formal thought disorder, and she denied suicidal ideation. While relapse of obsessive–compulsive disorder or severe health anxiety was considered, the persistence and rigidity of her beliefs supported a diagnosis of delusional disorder, somatic type.

**Results:**

This case illustrates how a plausible environmental event may catalyse the consolidation of somatic delusion, marking a transition from threat-based anxiety and obsessional monitoring to psychotic-level conviction. It highlights the phenomenological continuity between obsession and delusion and exposes the limitations of rigid diagnostic boundaries when symptoms evolve across domains. Clinically, the case underscores the profound functional impairment associated with somatic delusions and the therapeutic challenges posed by limited insight and partial engagement with treatment.

**Conclusion:**

This presentation emphasises the need for integrative formulations that account for environmental triggers, affective vulnerability, and evolving belief structures. It supports a dimensional understanding of psychopathology and the value of multidisciplinary approaches in assessing and managing complex somatic delusional states.

